# Estradiol and intrauterine device treatment for moderate and severe intrauterine adhesions after transcervical resection

**DOI:** 10.1186/s12905-022-01940-6

**Published:** 2022-08-29

**Authors:** Yun Sun, Xiuying Chen, Zhida Qian, Lili Cao, Sifeng Zhan, Lili Huang

**Affiliations:** 1 Women’s Hospital, Zhejiang University School of Medicine, 1 Xueshi Road, Hangzhou, 310006 Zhejiang Province People’s Republic of China; 2 The Affiliated Taian City Central Hospital of Qingdao University, Tai’an, 271000 Shandong Province People’s Republic of China; 3 The Fourth Affiliated Hospital of Zhejiang University School of Medicine, Zhejiang Province, Hangzhou, 310014 People’s Republic of China; 4 Taian Maternal and Child Health Hospital, Tai’an, 271025 Shandong Province People’s Republic of China

**Keywords:** Intrauterine adhesions, Transcervical resection of adhesion (TCRA), Intrauterine device, Estrogen

## Abstract

**Objective:**

To explore the effect of 4 mg/day, 6 mg/day, and 8 mg/day estradiol alone or in combination with an intrauterine device (IUD) in patients with moderate and severe intrauterine adhesion (IUA) after transcervical resection of adhesion (TCRA).

**Methods:**

Patients with moderate or severe IUA who reived 4 mg/day, 6 mg/day, and 8 mg/day estradiol alone or in combination with an intrauterine device (IUD) after TCRA in Women’s Hospital, Zhejiang University School of Medicine, from March 2014 to December 2014 were enrolled in this retrospective case–control study. In group A, 14 patients received estradiol 4 mg/day + IUD after the first operation; in group B, 29 patients (group B0) received estradiol 6 mg/day after the first operation, and 73 patients (group B1) received estradiol 6 mg/day + IUD; in group C, 14 patients received estradiol 8 mg/day + IUD after the first operation. Referring to ESGE's IUA diagnostic classification method, 72 patients had moderate adhesion, and 58 cases had severe adhesion. Outpatient follow-up was performed at 1 and 23 months and after 1 year. The postoperative menstrual improvement, uterine cavity recovery, drug side effects at two to three months, and pregnancy situation at one year were recorded.

**Results:**

There were no significant differences in age, BMI, and previous intrauterine operation times between the 3 groups (all p > 0.05). Compared with Group A, more patients in group C had severe IUA (p = 0.008). In addition, there were no differences in menstrual recovery, uterine cavity recovery, and pregnancy in one year between the 3 groups (p > 0.05) and between groups B_0_ and B_1_ (p > 0.05). In group B1, 51 (69.86%) patients had IUD incarceration.

**Conclusion:**

This data suggests that 4 mg/d doses of estrogen may have the same effect in improving the menstrual condition, uterine cavity morphology, and reproductive ability compared to a higher dosage (6 mg/day estrogen and 8 mg/day). In addition, the placement of IUD in the uterine cavity during TCRA may cause IUD incarceration, and the treatment results for the prevention of IUA are not better than without IUD.

## Introduction

Intrauterine adhesion (IUA) is a condition characterized by scar tissue in the endometrium caused by trauma, infection, and other factors, such as inappropriate intrauterine surgery. The main clinical manifestations of IUA are hypomenorrhea, recurrent abortion, amenorrhea, periodic hypogastralgia, infertility, and placental implantation [1]. With the increase in intrauterine operations and the popularity of hysteroscopic techniques, the incidence of IUA has been rising [2]. Currently, transcervical resection of adhesion (TCRA) by hysteroscopy is considered the main treatment for IUA. However, this treatment is associated with a high recurrence rate [3]. The rate of re-adhesion among moderate IUA patients and severe IUA patients after TCRA is 30% and 66.7%, respectively [4]. Therefore, the prevention of recurrence of adhesion after TCRA has become a hot topic among clinicians.

The two most important factors in preventing adhesion recurrence are physical barriers that maintain uterine morphology and estrogen-promoting endometrial growth [5, 6]. Estrogen is an important steroid hormone that can promote endometrial regeneration and capillary formation after menstruation [7]. Estrogen therapy is commonly used as an ancillary treatment after adhesiolysis since it accelerates the regeneration of the endometrial layer and prevents recurrent adhesions [8].

An intrauterine device (IUD), widely used as a birth control method, can reduce adhesion formation. Most IUDs are placed in the uterine cavity for 2–3 months in TCRA combined with estrogen or sodium hyaluronate. A study that used IUD alone as an ancillary treatment reported an overall natural pregnancy rate and live birth rate of 47.2% and 28.0%, respectively, after IUA separation surgery [9]. However, there is still no consensus on the specific estrogen dose and its physical relationship with IUD.

This study aimed to explore the effect of different doses of estrogen in combination with IUD after TCRA for treating moderate and severe IUA.

## Materials and methods

### Study design and participants

Patients with moderate or severe IUA who underwent TCRA in Women’s Hospital, Zhejiang University, School of Medicine between March 2014 and December 2014 were enrolled in this study. Inclusion criteria were: (1) women aged 18 to 40 years; (2) moderate-to-severe intrauterine adhesion (AFS score ≥ 5) [10]; (3) no previous history of adhesiolysis; (4) with a history of a second-look hysteroscopy; (5) desired future fertility. Exclusion criteria were: (1) genital tract malformations; (2) acute infection.

In group A, 14 patients received estradiol 4 mg/day + IUD after the first operation; in group B, 29 patients (group B0) received estradiol 6 mg/day after the first operation, and 73 patients (group B1) received estradiol 6 mg/day + IUD; in group C, 14 patients received estradiol 8 mg/day + IUD after the first operation. Referring to ESGE's IUA diagnostic classification method, 72 patients had moderate adhesion, and 58 cases had severe adhesion.

All included patients underwent intrauterine adhesion surgery for the first time. In addition, all patients underwent hysteroscopy to evaluate the adhesion recovery 2–3 months after surgery; at that time, IUA was removed.

This study was approved by the ethics committee of the Women's Hospital, Zhejiang University School of Medicine (IRB-20200100-R). The informed consent was waived as this was a retrospective study. All methods were carried out in accordance with relevant guidelines and regulations or the declaration of Helsinki.

### Data collection

Clinical data included 2 indicators for grouping (dose of estriol and whether IUD was used), 4 indicators for general characteristics (age, BMI, previous intrauterine operation times, and degree of intrauterine adhesion), 3 indicators for the results (menstrual recovery, uterine cavity recovery and pregnancy in one year) and adverse events related to treatments during follow-up. All patients were followed up after surgery. The liver and kidney function and normal blood coagulation were reviewed monthly until the end of estradiol therapy. In addition, transvaginal ultrasound was performed at 1, 2, and 3 months and 1 year after the first operation. The postoperative menstrual improvement, uterine cavity recovery, drug side effects at two to three months, and pregnancy situation at one year were recorded.

### Statistical analysis

SPSS 20.0 was employed for data analysis. P-value indicated the two-sided probability, and P-value < 0.05 was considered to be statistically significant. All patients were subjected to the Kolmogorov–Smirnov test (normal distribution test) together with the variance homogeneity test (Levene’s Test). For normally distributed data with homogenous variance, the independent sample t-test was used, and values are presented as means ± standard deviation. Meanwhile, data regarding the composition ratio were measured by the chi-square test (χ^2^ test), and values are presented as cases (percentages).

## Results

### Follow-up

Two to three months after surgery, patients underwent hysteroscopy, and IUDs were taken out. The patients were followed up for 1, 2–3 months, and 1 year. The postoperative menstrual improvement, uterine cavity recovery, drug side effects at two to three months, and pregnancy situation at one year were recorded. Twelve patients were lost to follow-up: 8 canceled their birth plans, while the other 4 went to other hospitals for treatment.

### General characteristics of the patients in different groups

The general characteristics of the patients are shown in Table 1. There was no significant difference in age (Group A vs. Group B, p = 0.302; Group A vs. Group C, p = 0.592; Group B vs. Group C, p = 0.742), BMI (Group A vs. Group B, p = 0.619; Group A vs. Group C, p = 0.057; Group B vs. Group C, p = 0.742) and previous intrauterine operation time (Group A vs. Group B, p = 0.145; Group A vs. Group C, p = 0.853; Group B vs. Group C, p = 0.072) between groups. Compared with Group A, more patients in group C had severe IUA (p = 0.008).

**Table 1:** General characteristics of patients in different groups

Project	Group A (N=14)	Group B (N=102)	Group C (N=14)
Age, years	31.42 ± 4.60	30.07 ± 4.60	30.50 ± 4.45
BMI, kg/m^2^	23.40 ± 1.45	23.15 ± 1.77	22.26 ± 1.56
Previous intrauterine operations, times	3.00 ± 2.00	2.40 ± 1.34	3.14 ± 2.03
Degree of intrauterine adhesion, cases			
Moderate IUA	11 (78.57%)	57 (55.88%)	4 (28.57%)*
Severe IUA	3 (21.43%)	45 (44.12%)	10 (71.43%)

*Compared with Group A, p value < 0.05

### The treatment results

#### Treatment results in patients with moderate or severe IUA

Treatment results in patients with moderate or severe IUA are shown in Table 2. There were no differences in menstrual recovery (p = 0.691) and uterine cavity recovery (p = 0.582) between patients with moderate and severe IUA. Yet, severe IUA patients had a lower one-year pregnancy rate compared to moderate IUA patients (p = 0.046).

**Table 2:** Comparison of the results in moderate and severe IUA patients

	Moderate IUA N=72	Severe IUA N=58	p value
Menstrual recovery, cases	51 (70.83%)	35 (60.34%)	0.691
Uterine cavity recovery, cases	44 (61.11%)	35 (60.34%)	0.582
Pregnancy in one year, cases	25 (34.72%)	11 (18.97%)	0.046

#### The results of patients treated with different doses of estradiol and IUD

The results of group A, group B1, and group C are shown in Table 3. There was no significant difference in menstrual recovery among moderate IUA patients (Group A vs. Group B_1_, p = 0.484; Group A vs. Group C, p = 0.680; Group B_1_ vs. Group C, p = 0.978) and among severe IUA patients (Group A vs. Group B_1_, p = 0.233; Group A vs. Group C, p = 0.416; Group B_1_ vs. Group C, p = 0.654), and the uterine cavity recovery among moderate IUA patients (Group A vs. Group B_1_, p = 0.364; Group A vs. Group C, p = 0.310; Group B_1_ vs. Group C, p = 0.077) and severe IUA patients (Group A vs. Group B_1_, p = 0.336; Group A vs. Group C, p = 0.252; Group B_1_ vs. Group C, p = 0.634). Moreover, there was no significant difference in pregnancy rate at one year among moderate IUA patients (Group A vs. Group B_1_, p = 0.564; Group A vs. Group C, p = 0.475; Group B_1_ vs. Group C, p = 0.663) and severe IUA patients (Group A vs. Group B_1_, p = 0.343; Group A vs. Group C, p = 0.400; Group B_1_ vs. Group C, p = 0.815).

**Table 3:** Comparison of the result of patients using different concentrations of estriol plus IUD

	Group A (N=14)	Group B1 (N=73)	Group C (N=14)
Menstrual recovery, cases (%)			
Moderate	7 (63.63%)	29 (74.36%)	3 (75.00%)
Severity	1 (33.33%)	23 (67.65%)	6 (60.00%)
Uterine cavity recovery, cases (%)			
Moderate	6 (54.54%)	27 (69.23%)	1 (25.00%)
Severe	1 (33.33%)	21 (61.76%)	7 (70.00%)
Pregnancy in one year, cases (%)			
Moderate	5 (45.45%)	14 (35.90%)	1 (25.00%)
Severity	0 (0%)	8 (23.53%)	2 (20.00%)

During follow-up, 1 patient (1%) in group A, 1 patient (1.4%) in group B1, and 2 patients (14.3%) in group C had mild nausea, which was resolved after 5–7 days. One patient in group C had mild breast pain in the premenstrual period but no abnormal findings in the breast B ultrasound. All these patients continued using the medication.

#### The results of patients treated with 6 mg/day estriol with or without IUD

The results of the B0 and B1 groups are shown in Table 4. There was no difference in menstrual recovery among moderate IUA patients (p = 0.548) and severe IUA patients from the two groups (p = 0.187). In addition, there was no significant difference in uterine cavity recovery among moderate IUA patients (p = 0.315) and severe IUA patients in the two groups (p = 0.671). Also, there was no significant difference in pregnancy rate at one year among moderate IUA patients (p = 0.546) and severe IUA patients in the two groups (p = 0.298).

**Table 4:** Comparison of results between groups B0 and B1

	Group B0 (N = 29)	Group B1 (N = 73)	P value
Menstrual recovery, cases (%)			
Moderate	12 (66.67%)	29 (74.36%)	0.548
Severe	5 (45.45%)	23 (67.65%)	0.187
Uterine cavity recovery, cases (%)			
Moderate	10 (55.56%)	27(69.23%)	0.315
Severe	6 (54.55%)	21 (61.76%)	0.671
Pregnancy in 1 year, cases (%)			
Moderate	5 (27.78%)	14 (35.90%)	0.546
Severe	1 (9.09%)	8 (23.53%)	0.298

During follow-up, one patient (3.4%) in the B0 group developed mild appetite loss that spontaneously improved after 5 days without treatment. In group B1, 51 (69.86%) patients had IUD incarceration; among them, 2 patients had bloody leucorrhea and 2 developed back pain. These four patients underwent hysteroscopy to remove IUDs in the second month after the first surgery.

## Discussion

Some studies found that estrogen therapy can induce neointimal growth, thus preventing adhesion recurrence and increasing the amount of menstruation [11, 12]. Other studies suggested that low-dose estrogen status affects endometrial regeneration [13, 14]. Estradiol valerate is an estradiol prodrug used to treat some effects of menopause, hypoestrogenism, and androgen dependant carcinoma. It has no associations with common gastrointestinal reactions or potential teratogenicity [14]. However, there is still no universal consensus on standard doses. The dosage of estradiol valerate ranges from 1 to 18 mg/day.

In the present study, we found that estradiol valerate after IUA separation could achieve a good pregnancy result. Yet, we found no difference in the efficacy when different estradiol concentrations were used (4 mg/day, 6 mg/day, and 8 mg/day). There was probably not enough residual endometrial area and endometrial regeneration function in patients with IUA. When the estrogen receptor in the endometrial is saturated, the therapeutic effect of estrogen cannot be improved. Some studies have found that in IUA patients with intrauterine fibrosis, the effective contraction of the myometrium is severely limited, which in turn inhibits the diffusion of steroidal estrogen into the intimal layer [15–17], thus negatively affecting the treatment [18]. It is also well known that a higher dosage of estrogen is positively correlated with adverse drug reactions, such as liver and kidney dysfunction, coagulopathy, and even thrombosis [19–21]. In this study, we found no difference in the therapeutic results between groups A, B_1,_ and C, which suggested that 4 mg/d doses of estrogen may have the same effect in improving the menstrual condition, uterine cavity morphology, and reproductive ability compared to a higher dosage.

In this study, there was no significant difference in the therapeutic effect between the B0 and B1 groups, suggesting that simultaneous placement of IUD in TCRA does not improve the therapeutic effect. In addition, the local inflammatory reaction of the uterine cavity caused by copper-containing IUD may disturb the growth of normal endometrium. Therefore, although many reports have produced good results and IUD has been widely used, the placement of IUD after TRCA remains controversial. Also, IUD has been associated with several side effects, such as infection, incarceration, rupture, and difficulty removing the implant.

This study has a few limitations. Firstly, it was a non-randomized and retrospective study. Secondly, other effective methods to treat IUA, including stem cells and hyaluronan gel, were not considered. Thirdly, this study was a retrospective study with a short fellow up. The involved cases were selected strictly according to the inclusion and exclusion criteria, and 12 cases were lost to follow-up, resulting in a small number of total cases and unbalanced cases among the three groups.

In conclusion, our data suggested that 4 mg/day doses of estrogen may have the same effect in improving the menstrual condition, uterine cavity morphology, and reproductive ability compared to a higher dosage (6 mg/day estrogen and 8 mg/day). In addition, the placement of IUD in the uterine cavity during TCRA may cause IUD incarceration, and the treatment results for the prevention of IUA are not better than without IUD. Therefore, a larger sample, multi-center RCT study is needed to further explore the application of different treatment methods after TCRA.

## Data Availability

All data generated or analyzed during this study are included in this published article.
